# La_5_Zn_2_Sn

**DOI:** 10.1107/S1600536811042413

**Published:** 2011-10-22

**Authors:** Igor Oshchapovsky, Volodymyr Pavlyuk, Grygoriy Dmytriv, Igor Chumak, Helmut Ehrenberg

**Affiliations:** a Department of Inorganic Chemistry, Ivan Franko Lviv National University, Kyryla i Mefodia 6,79005 Lviv, Ukraine; b Institute for Complex Materials, IFW Dresden, Helmholz Strasse 20, D-01069 Dresden, Germany; c Karlsruhe Institute of Technology (KIT), Institute for Applied Materials (IAM), Hermann-von-Helmholtz-Platz 1, D-76344 Eggenstein-Leopoldshafen, Germany

## Abstract

A single crystal of penta­lanthanum dizinc stannide, La_5_Zn_2_Sn, was obtained from the elements in a resistance furnace. It belongs to the Mo_5_SiB_2_ structure type, which is a ternary ordered variant of the Cr_5_B_3_ structure type. The space is filled by bicapped tetra­gonal anti­prisms from lanthanum atoms around tin atoms sharing their vertices. Zinc atoms fill voids between these bicapped tetra­gonal anti­prisms. All four atoms in the asymmetric unit reside on special positions with the following site symmetries: La1 (..*m*); La2 (4/*m*..); Zn (*m*.2*m*); Sn (422).

## Related literature

For general background to {Tb,La}–Zn–{Sn,Pb} ternary systems, see: Manfrinetti & Pani, (2005 ▶); Oshchapovsky *et al.* (2010 ▶, 2011 ▶); Pavlyuk *et al.* (2009 ▶). For related structures, see: Bertaut (1953 ▶). For isotypic structures, see: Aronsson (1958 ▶).

## Experimental

### 

#### Crystal data

La_5_Zn_2_Sn
*M* *_r_* = 944.04
Tetragonal, 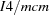

*a* = 8.3277 (12) Å
*c* = 14.334 (3) Å
*V* = 994.1 (3) Å^3^
*Z* = 4
Mo *K*α radiation
μ = 28.10 mm^−1^
*T* = 293 K
0.04 × 0.04 × 0.01 mm

#### Data collection

Bruker APEXII CCD diffractometer
Absorption correction: multi-scan (*SADABS*; Bruker, 2004 ▶) *T*_min_ = 0.340, *T*_max_ = 0.765
9291 measured reflections
419 independent reflections
346 reflections with *I* > 2σ(*I*)
*R*_int_ = 0.091

#### Refinement

*R*[*F* ^2^ > 2σ(*F* ^2^)] = 0.027
*wR*(*F* ^2^) = 0.056
*S* = 1.14
419 reflections
14 parameters
Δρ_max_ = 1.60 e Å^−3^
Δρ_min_ = −1.59 e Å^−3^

### 

Data collection: *APEX2* (Bruker, 2004 ▶); cell refinement: *SAINT* (Bruker, 2004 ▶); data reduction: *SAINT*; program(s) used to solve structure: *SHELXS97* (Sheldrick, 2008 ▶); program(s) used to refine structure: *SHELXL97* (Sheldrick, 2008 ▶) and *JANA2006* (Petricek *et al.*, 2006 ▶); molecular graphics: *DIAMOND* (Brandenburg, 2006 ▶) and *VESTA* (Momma & Izumi, 2008 ▶); software used to prepare material for publication: *publCIF* (Westrip, 2010 ▶).

## Supplementary Material

Crystal structure: contains datablock(s) I, global. DOI: 10.1107/S1600536811042413/ru2016sup1.cif

Structure factors: contains datablock(s) I. DOI: 10.1107/S1600536811042413/ru2016Isup2.hkl

Additional supplementary materials:  crystallographic information; 3D view; checkCIF report

## Figures and Tables

**Fig. 1.**
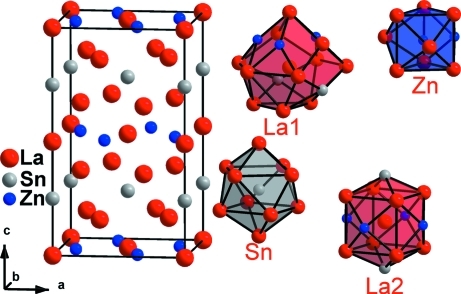
Unit cell projection and coordination polyhedra in the La5Zn2Sn compound

**Fig. 2.**
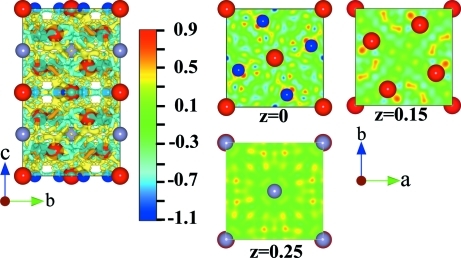
Isosurface drawn at 0.308 e/Å3 and sections of difference Fourier map after the refinement of crystal structure of the La5Zn2Sn compound

## supplementary crystallographic information

### Comment

Ternary compounds formed by rare-earth, transition metal and d-metal often have interesting physical and chemical properties e. g. strong ferromagnetism, hydrogen storage capabilities and so on. The systematic investigation of the components interaction in the {Tb, La}-Zn-{Sn,Pb} ternary systems can lead to development of functional materials (for crystal structures of ternary compounds see: TbZnSn –Manfrinetti & Pani, (2005), Pavlyuk *et al.*, (2009), TbZnSn_2_ - Pavlyuk *et al.*, (2009), Tb_13_ZnSn_13_*-*Oshchapovsky *et al.*, (2010) and LaZn_12.37_*-*Oshchapovsky *et al.*, (2011)).

The title compound crystallizes in Mo_5_SiB_2_ (Aronsson, 1958) structure type which is an ordered superstructure of Cr_5_B_3_ type (Bertaut, 1953). Unit cell projection together with coordination polyhedra are given in Fig.1. Coordination polyhedra of the La1 atoms are 16- vertex polyhedra. Sn atoms are surrounded by ten neighbours forming bicapped tetragonal antiprism. La2 atoms are enclosed into trigon - tetrahexahedron with CN=14. And coordination polyhedra of the Zn atoms are bicapped trigonal prisms with CN=9. Coordination polyhedra of Sn atoms share their vertices forming three dimensional framework. The voids in this framework are filled by zinc atoms. (See graphical abstract).

The way of bond formation in this compound was assumed using only X-ray diffraction data. Further structure refinement was carried out by means of Jana2006 software package using anharmonic ADP for La1 and Zn atoms. Anharmonic displacement parameters for other atoms were not refined because in case of their refinement their standard deviations were larger than obtained values. As the result we gained lower absolute values of peak and hole in the difference Fourier map (1.02 and -1.17 e Å^-3^ respectively). The resulting isosurface drawn at the level 0.308 e/Å^3^ and sections of difference Fourier map are given in Fig. 2. These maps and sections are noisy but some trends in location of positive and negative regions can be noticed. Positive residual electron density is mostly situated around zinc atoms and near layers made of tin atoms. Negative residual density is mostly located between lanthanum atoms which means that lanthanum atoms donate their electrons to zinc and tin atoms. Similar behaviour of lanthanum atoms can be observed in the LaZn_12.37_ compound using electronic structure calculations (See Oshchapovsky *et al.*, (2011)). As a conclusion this compound besides dominate metallic bonding has a weak ionic interaction between lanthanum and zinc and tin atoms.

### Experimental

Small good quality single-crystal of title compound was isolated from alloy with composition La_7_ZnSn_2_ during systematic investigation of lanthanum-rich region of La—Zn—Sn ternary system. The samples with high lanthanum contents were prepared by melting of pieces of pure metals in evacuated quartz ampoule with subsequent annealing at 600 ^0^C for 30 days. Further phase analysis showed the existence of title compound in sample with composition La_7_ZnSn_2_ as well as in the other lanthanum-rich ternary alloys. However they were non equilibrium.

### Crystal data

**Table:**

La_5_Zn_2_Sn	*D*_x_ = 6.308 Mg m^−^^3^
*M**_r_* = 944.04	Mo *K*α radiation, λ = 0.71073 Å
Tetragonal, *I*4/*m**c**m*	Cell parameters from 1223 reflections
Hall symbol: -I 4 2c	θ = 5.7–26.1°
*a* = 8.3277 (12) Å	µ = 28.10 mm^−^^1^
*c* = 14.334 (3) Å	*T* = 293 K
*V* = 994.1 (3) Å^3^	Plate, grey
*Z* = 4	0.04 × 0.04 × 0.01 mm
*F*(000) = 1580.0	

### Data collection

**Table:**

Bruker APEXII CCD diffractometer	419 independent reflections
Radiation source: sealed tube	346 reflections with *I* > 2σ(*I*)
graphite	*R*_int_ = 0.091
Detector resolution: 8.366 pixels mm^-1^	θ_max_ = 30.1°, θ_min_ = 2.8°
φ and ω scans	*h* = −11→11
Absorption correction: multi-scan (*SADABS*; Bruker, 2004)	*k* = −11→11
*T*_min_ = 0.340, *T*_max_ = 0.765	*l* = −20→19
9291 measured reflections	

### Refinement

**Table:**

Refinement on *F*^2^	0 restraints
Least-squares matrix: full	Primary atom site location: structure-invariant direct methods
*R*[*F*^2^ > 2σ(*F*^2^)] = 0.027	Secondary atom site location: difference Fourier map
*wR*(*F*^2^) = 0.056	*w* = 1/[σ^2^(*F*_o_^2^) + (0.0144*P*)^2^ + 16.4322*P*] where *P* = (*F*_o_^2^ + 2*F*_c_^2^)/3
*S* = 1.14	(Δ/σ)_max_ = 0.004
419 reflections	Δρ_max_ = 1.60 e Å^−^^3^
14 parameters	Δρ_min_ = −1.59 e Å^−^^3^

### Special details

**Table:**

Geometry. All e.s.d.'s (except the e.s.d. in the dihedral angle between two l.s. planes) are estimated using the full covariance matrix. The cell e.s.d.'s are taken into account individually in the estimation of e.s.d.'s in distances, angles and torsion angles; correlations between e.s.d.'s in cell parameters are only used when they are defined by crystal symmetry. An approximate (isotropic) treatment of cell e.s.d.'s is used for estimating e.s.d.'s involving l.s. planes.
Refinement. Refinement of *F*^2^ against ALL reflections. The weighted *R*-factor *wR* and goodness of fit *S* are based on *F*^2^, conventional *R*-factors *R* are based on *F*, with *F* set to zero for negative *F*^2^. The threshold expression of *F*^2^ > σ(*F*^2^) is used only for calculating *R*-factors(gt) *etc*. and is not relevant to the choice of reflections for refinement. *R*-factors based on *F*^2^ are statistically about twice as large as those based on *F*, and *R*- factors based on ALL data will be even larger.

### Fractional atomic coordinates and isotropic or equivalent isotropic displacement parameters (Å^2^)

**Table:**

	*x*	*y*	*z*	*U*_iso_*/*U*_eq_	
La1	0.66685 (5)	0.16685 (5)	0.14853 (4)	0.02054 (16)	
La2	0.0000	0.0000	0.0000	0.0357 (3)	
Zn	0.12383 (12)	0.62383 (12)	0.0000	0.0139 (3)	
Sn	0.0000	0.0000	0.2500	0.0145 (2)	

### Atomic displacement parameters (Å^2^)

**Table:**

	*U*^11^	*U*^22^	*U*^33^	*U*^12^	*U*^13^	*U*^23^
La1	0.01937 (19)	0.01937 (19)	0.0229 (3)	0.00430 (19)	0.000	0.000
La2	0.0291 (4)	0.0291 (4)	0.0490 (8)	0.000	0.000	0.000
Zn	0.0123 (4)	0.0123 (4)	0.0171 (7)	0.0013 (5)	0.000	0.000
Sn	0.0129 (3)	0.0129 (3)	0.0177 (5)	0.000	0.000	0.000

### Geometric parameters (Å, °)

**Table:**

La1—Zn^i^	3.2436 (9)	La2—La1^xvii^	3.7630 (6)
La1—Zn^ii^	3.2436 (9)	Zn—Zn^x^	2.917 (3)
La1—Zn^iii^	3.2573 (12)	Zn—La1^xviii^	3.2436 (9)
La1—Sn^iv^	3.4268 (5)	Zn—La1^xvi^	3.2436 (9)
La1—Sn^v^	3.4268 (5)	Zn—La1^xix^	3.2436 (9)
La1—La1^vi^	3.5068 (12)	Zn—La1^xiv^	3.2436 (9)
La1—La2^iv^	3.7630 (6)	Zn—La1^xx^	3.2573 (12)
La1—La2^vii^	3.7630 (6)	Zn—La1^iii^	3.2573 (12)
La1—La1^viii^	3.9301 (12)	Zn—La2^xxi^	3.2980 (8)
La2—Zn^ix^	3.2980 (8)	Zn—La2^vii^	3.2980 (8)
La2—Zn^ii^	3.2980 (8)	Sn—La1^xxii^	3.4268 (5)
La2—Zn^x^	3.2980 (8)	Sn—La1^xvii^	3.4268 (5)
La2—Zn^xi^	3.2980 (8)	Sn—La1^xiv^	3.4268 (5)
La2—Sn^xii^	3.5835 (7)	Sn—La1^v^	3.4268 (5)
La2—Sn	3.5835 (7)	Sn—La1^xxiii^	3.4268 (5)
La2—La1^xiii^	3.7630 (6)	Sn—La1^xxiv^	3.4268 (5)
La2—La1^xiv^	3.7630 (6)	Sn—La1^viii^	3.4268 (5)
La2—La1^xv^	3.7630 (6)	Sn—La1^xxv^	3.4268 (5)
La2—La1^viii^	3.7630 (6)	Sn—La2^xxvi^	3.5835 (7)
La2—La1^xvi^	3.7630 (6)		
			
Zn^i^—La1—Zn^ii^	53.44 (5)	Zn^ii^—La2—La1^xvi^	54.461 (17)
Zn^i^—La1—Zn^iii^	91.69 (2)	Zn^x^—La2—La1^xvi^	54.208 (18)
Zn^ii^—La1—Zn^iii^	91.69 (2)	Zn^xi^—La2—La1^xvi^	125.792 (18)
Zn^i^—La1—Sn^iv^	93.75 (2)	Sn^xii^—La2—La1^xvi^	55.545 (10)
Zn^ii^—La1—Sn^iv^	146.93 (3)	Sn—La2—La1^xvi^	124.455 (10)
Zn^iii^—La1—Sn^iv^	93.506 (15)	La1^xiii^—La2—La1^xvi^	111.09 (2)
Zn^i^—La1—Sn^v^	146.93 (3)	La1^xiv^—La2—La1^xvi^	68.91 (2)
Zn^ii^—La1—Sn^v^	93.75 (2)	La1^xv^—La2—La1^xvi^	71.332 (10)
Zn^iii^—La1—Sn^v^	93.506 (15)	La1^viii^—La2—La1^xvi^	108.668 (10)
Sn^iv^—La1—Sn^v^	118.450 (18)	Zn^ix^—La2—La1^xvii^	54.461 (17)
Zn^i^—La1—La1^vi^	151.98 (2)	Zn^ii^—La2—La1^xvii^	125.539 (17)
Zn^ii^—La1—La1^vi^	151.98 (2)	Zn^x^—La2—La1^xvii^	125.792 (18)
Zn^iii^—La1—La1^vi^	96.86 (3)	Zn^xi^—La2—La1^xvii^	54.208 (18)
Sn^iv^—La1—La1^vi^	59.225 (9)	Sn^xii^—La2—La1^xvii^	124.455 (10)
Sn^v^—La1—La1^vi^	59.225 (9)	Sn—La2—La1^xvii^	55.545 (10)
Zn^i^—La1—La2^iv^	55.564 (18)	La1^xiii^—La2—La1^xvii^	68.91 (2)
Zn^ii^—La1—La2^iv^	97.94 (3)	La1^xiv^—La2—La1^xvii^	111.09 (2)
Zn^iii^—La1—La2^iv^	55.477 (10)	La1^xv^—La2—La1^xvii^	108.668 (10)
Sn^iv^—La1—La2^iv^	59.571 (13)	La1^viii^—La2—La1^xvii^	71.332 (10)
Sn^v^—La1—La2^iv^	146.931 (17)	La1^xvi^—La2—La1^xvii^	180.000 (16)
La1^vi^—La1—La2^iv^	108.903 (17)	Zn^x^—Zn—La1^xviii^	63.28 (2)
Zn^i^—La1—La2^vii^	97.94 (3)	Zn^x^—Zn—La1^xvi^	63.28 (2)
Zn^ii^—La1—La2^vii^	55.564 (18)	La1^xviii^—Zn—La1^xvi^	74.58 (3)
Zn^iii^—La1—La2^vii^	55.477 (10)	Zn^x^—Zn—La1^xix^	63.28 (2)
Sn^iv^—La1—La2^vii^	146.931 (17)	La1^xviii^—Zn—La1^xix^	82.05 (3)
Sn^v^—La1—La2^vii^	59.571 (13)	La1^xvi^—Zn—La1^xix^	126.56 (5)
La1^vi^—La1—La2^vii^	108.903 (17)	Zn^x^—Zn—La1^xiv^	63.28 (2)
La2^iv^—La1—La2^vii^	102.966 (18)	La1^xviii^—Zn—La1^xiv^	126.56 (5)
Zn^i^—La1—La1^viii^	52.712 (14)	La1^xvi^—Zn—La1^xiv^	82.05 (3)
Zn^ii^—La1—La1^viii^	52.712 (14)	La1^xix^—Zn—La1^xiv^	74.58 (3)
Zn^iii^—La1—La1^viii^	139.19 (2)	Zn^x^—Zn—La1^xx^	139.19 (2)
Sn^iv^—La1—La1^viii^	106.604 (9)	La1^xviii^—Zn—La1^xx^	140.29 (2)
Sn^v^—La1—La1^viii^	106.604 (9)	La1^xvi^—Zn—La1^xx^	140.29 (2)
La1^vi^—La1—La1^viii^	123.951 (19)	La1^xix^—Zn—La1^xx^	84.909 (18)
La2^iv^—La1—La1^viii^	105.084 (8)	La1^xiv^—Zn—La1^xx^	84.909 (18)
La2^vii^—La1—La1^viii^	105.084 (8)	Zn^x^—Zn—La1^iii^	139.19 (2)
Zn^i^—La1—La1^xxvii^	87.66 (2)	La1^xviii^—Zn—La1^iii^	84.909 (18)
Zn^ii^—La1—La1^xxvii^	113.473 (17)	La1^xvi^—Zn—La1^iii^	84.909 (18)
Zn^iii^—La1—La1^xxvii^	147.382 (8)	La1^xix^—Zn—La1^iii^	140.29 (2)
Sn^iv^—La1—La1^xxvii^	54.057 (10)	La1^xiv^—Zn—La1^iii^	140.29 (2)
Sn^v^—La1—La1^xxvii^	104.62 (2)	La1^xx^—Zn—La1^iii^	81.63 (4)
La1^vi^—La1—La1^xxvii^	70.91 (2)	Zn^x^—Zn—La2^xxi^	116.78 (2)
La2^iv^—La1—La1^xxvii^	98.861 (14)	La1^xviii^—Zn—La2^xxi^	70.228 (8)
La2^vii^—La1—La1^xxvii^	156.689 (9)	La1^xvi^—Zn—La2^xxi^	138.024 (12)
La1^viii^—La1—La1^xxvii^	60.762 (9)	La1^xix^—Zn—La2^xxi^	70.228 (8)
Zn^i^—La1—La1^xxii^	113.473 (17)	La1^xiv^—Zn—La2^xxi^	138.024 (12)
Zn^ii^—La1—La1^xxii^	87.66 (2)	La1^xx^—Zn—La2^xxi^	70.06 (2)
Zn^iii^—La1—La1^xxii^	147.382 (8)	La1^iii^—Zn—La2^xxi^	70.06 (2)
Sn^iv^—La1—La1^xxii^	104.62 (2)	Zn^x^—Zn—La2^vii^	116.78 (2)
Sn^v^—La1—La1^xxii^	54.057 (10)	La1^xviii^—Zn—La2^vii^	138.024 (12)
La1^vi^—La1—La1^xxii^	70.91 (2)	La1^xvi^—Zn—La2^vii^	70.228 (8)
La2^iv^—La1—La1^xxii^	156.689 (9)	La1^xix^—Zn—La2^vii^	138.024 (12)
La2^vii^—La1—La1^xxii^	98.861 (14)	La1^xiv^—Zn—La2^vii^	70.228 (8)
La1^viii^—La1—La1^xxii^	60.762 (9)	La1^xx^—Zn—La2^vii^	70.06 (2)
La1^xxvii^—La1—La1^xxii^	58.477 (18)	La1^iii^—Zn—La2^vii^	70.06 (2)
Zn^ix^—La2—Zn^ii^	180.0	La2^xxi^—Zn—La2^vii^	126.44 (4)
Zn^ix^—La2—Zn^x^	90.0	La1^xxii^—Sn—La1^xvii^	146.791 (18)
Zn^ii^—La2—Zn^x^	90.0	La1^xxii^—Sn—La1^xiv^	61.550 (18)
Zn^ix^—La2—Zn^xi^	90.0	La1^xvii^—Sn—La1^xiv^	129.77 (2)
Zn^ii^—La2—Zn^xi^	90.0	La1^xxii^—Sn—La1^v^	79.622 (8)
Zn^x^—La2—Zn^xi^	180.0	La1^xvii^—Sn—La1^v^	132.159 (17)
Zn^ix^—La2—Sn^xii^	90.0	La1^xiv^—Sn—La1^v^	71.89 (2)
Zn^ii^—La2—Sn^xii^	90.0	La1^xxii^—Sn—La1^xxiii^	129.77 (2)
Zn^x^—La2—Sn^xii^	90.0	La1^xvii^—Sn—La1^xxiii^	61.550 (18)
Zn^xi^—La2—Sn^xii^	90.0	La1^xiv^—Sn—La1^xxiii^	146.791 (18)
Zn^ix^—La2—Sn	90.0	La1^v^—Sn—La1^xxiii^	79.622 (8)
Zn^ii^—La2—Sn	90.0	La1^xxii^—Sn—La1^xxiv^	132.159 (17)
Zn^x^—La2—Sn	90.0	La1^xvii^—Sn—La1^xxiv^	79.622 (8)
Zn^xi^—La2—Sn	90.0	La1^xiv^—Sn—La1^xxiv^	79.622 (8)
Sn^xii^—La2—Sn	180.0	La1^v^—Sn—La1^xxiv^	61.550 (18)
Zn^ix^—La2—La1^xiii^	54.461 (17)	La1^xxiii^—Sn—La1^xxiv^	71.89 (2)
Zn^ii^—La2—La1^xiii^	125.539 (17)	La1^xxii^—Sn—La1^viii^	71.89 (2)
Zn^x^—La2—La1^xiii^	125.792 (18)	La1^xvii^—Sn—La1^viii^	79.622 (8)
Zn^xi^—La2—La1^xiii^	54.208 (18)	La1^xiv^—Sn—La1^viii^	79.622 (8)
Sn^xii^—La2—La1^xiii^	55.544 (10)	La1^v^—Sn—La1^viii^	146.791 (18)
Sn—La2—La1^xiii^	124.456 (10)	La1^xxiii^—Sn—La1^viii^	132.159 (17)
Zn^ix^—La2—La1^xiv^	125.539 (17)	La1^xxiv^—Sn—La1^viii^	129.77 (2)
Zn^ii^—La2—La1^xiv^	54.461 (17)	La1^xxii^—Sn—La1^xxv^	79.622 (8)
Zn^x^—La2—La1^xiv^	54.208 (18)	La1^xvii^—Sn—La1^xxv^	71.89 (2)
Zn^xi^—La2—La1^xiv^	125.792 (18)	La1^xiv^—Sn—La1^xxv^	132.159 (17)
Sn^xii^—La2—La1^xiv^	124.456 (10)	La1^v^—Sn—La1^xxv^	129.77 (2)
Sn—La2—La1^xiv^	55.544 (10)	La1^xxiii^—Sn—La1^xxv^	79.622 (8)
La1^xiii^—La2—La1^xiv^	180.000 (16)	La1^xxiv^—Sn—La1^xxv^	146.791 (18)
Zn^ix^—La2—La1^xv^	54.208 (18)	La1^viii^—Sn—La1^xxv^	61.550 (18)
Zn^ii^—La2—La1^xv^	125.792 (18)	La1^xxii^—Sn—La2^xxvi^	64.885 (10)
Zn^x^—La2—La1^xv^	54.461 (17)	La1^xvii^—Sn—La2^xxvi^	115.115 (10)
Zn^xi^—La2—La1^xv^	125.539 (17)	La1^xiv^—Sn—La2^xxvi^	115.115 (10)
Sn^xii^—La2—La1^xv^	55.544 (10)	La1^v^—Sn—La2^xxvi^	64.885 (10)
Sn—La2—La1^xv^	124.456 (10)	La1^xxiii^—Sn—La2^xxvi^	64.885 (10)
La1^xiii^—La2—La1^xv^	71.332 (10)	La1^xxiv^—Sn—La2^xxvi^	115.115 (10)
La1^xiv^—La2—La1^xv^	108.668 (10)	La1^viii^—Sn—La2^xxvi^	115.115 (10)
Zn^ix^—La2—La1^viii^	125.792 (18)	La1^xxv^—Sn—La2^xxvi^	64.885 (10)
Zn^ii^—La2—La1^viii^	54.208 (18)	La1^xxii^—Sn—La2	115.115 (10)
Zn^x^—La2—La1^viii^	125.539 (17)	La1^xvii^—Sn—La2	64.885 (10)
Zn^xi^—La2—La1^viii^	54.461 (17)	La1^xiv^—Sn—La2	64.885 (10)
Sn^xii^—La2—La1^viii^	124.456 (10)	La1^v^—Sn—La2	115.115 (10)
Sn—La2—La1^viii^	55.544 (10)	La1^xxiii^—Sn—La2	115.115 (10)
La1^xiii^—La2—La1^viii^	108.668 (10)	La1^xxiv^—Sn—La2	64.885 (10)
La1^xiv^—La2—La1^viii^	71.332 (10)	La1^viii^—Sn—La2	64.885 (10)
La1^xv^—La2—La1^viii^	180.000 (18)	La1^xxv^—Sn—La2	115.115 (10)
Zn^ix^—La2—La1^xvi^	125.539 (17)	La2^xxvi^—Sn—La2	180.0

Symmetry codes: (i) *y*, −*x*, −*z*; (ii) −*y*+1, *x*, *z*; (iii) −*x*+1, −*y*+1, −*z*; (iv) *x*+1, *y*, *z*; (v) −*x*+1/2, −*y*+1/2, −*z*+1/2; (vi) −*x*+3/2, −*y*+1/2, −*z*+1/2; (vii) −*x*+1/2, *y*+1/2, −*z*; (viii) −*x*+1, −*y*, *z*; (ix) *y*−1, −*x*, −*z*; (x) −*x*, −*y*+1, −*z*; (xi) *x*, *y*−1, *z*; (xii) −*x*, −*y*, −*z*; (xiii) −*y*, *x*−1, −*z*; (xiv) *y*, −*x*+1, *z*; (xv) *x*−1, *y*, −*z*; (xvi) *y*, −*x*+1, −*z*; (xvii) −*y*, *x*−1, *z*; (xviii) −*y*, *x*, −*z*; (xix) −*y*, *x*, *z*; (xx) −*x*+1, −*y*+1, *z*; (xxi) *x*, *y*+1, *z*; (xxii) −*y*+1/2, *x*−1/2, −*z*+1/2; (xxiii) *y*−1/2, −*x*+1/2, −*z*+1/2; (xxiv) *x*−1, *y*, *z*; (xxv) *x*−1/2, *y*−1/2, −*z*+1/2; (xxvi) −*x*, *y*, −*z*+1/2; (xxvii) *y*+1/2, −*x*+1/2, −*z*+1/2.
